# Tenement Houses

**Published:** 1919-09-13

**Authors:** 


### TENEMENT HOUSES.

### I. Some Drastic Changes Called For.

When one speaks of tenement houses the mind naturally pictures that atrocious compilation known as the "block dwelling," but, like everything to which a certain epithet is given, there are "tenement houses and tenement houses," ranging from the block of five to ten storeys high down to the modest building of two storeys—composite buildings which were all intended by the architect to be more or less self-contained. Then you have the ordinary many-roomed private dwelling-houses, originally intended for one family, cut up and partitioned into separate tenements of from one to three or four rooms, but which can rarely be styled self-contained by the greatest stretch of imagination. This latter style of tenement house should never be permitted, except in an emergency, since for obvious reasons it is generally a hotbed of disease and vice.

### Obvious Limitations.

The various tenements cannot be effectively partitioned or separated, since the rooms were originally part of a design which had for its object the easy communication of rooms with one another either direct or by the intervention of a common passage or staircase, and it stands to reason that these communications cannot be altered or dispensed with except by a complete gutting of the interior, which costs almost as much as a new house and is never satisfactory. The result of such manufactured tenements is that there is of necessity a very free intercommunication of the inhabitants, which means facilities for the spread of infectious disease, as well as vice, and finally leading to the avoidance of the neighbourhood by the better class of inhabitants and the eventual handing of them over to utterly thriftless and criminal classes.

### Origin of Tenement Dwellings.

It is difficult to say when the first blocks of dwellings were built, but probably the oldest existing ones will not date back beyond the middle of last century. The reason for their existence is no doubt the rise in the value of land in large towns owing to the demand for building sites, and it was found that it was a good commercial speculation to erect working-class houses on top of one another where lateral expansion was impossible. In these earlier types too much attention was paid to the utilising of space and too little to the ordinary amenities of family life, the result being that the *whole accommodation* of a tenement may range from a single bedroom to a suite of two to four rooms, one of which can be used as a kitchen or sitting-room and the remainder as bedrooms. There is only one w.c. for every two or three tenements, no scullery or bathroom, and either no accommodation for washing or else a common wash-house provided on the flat roof, where the occupants have to carry up their coal and occasionally the water, the result being that they prefer to do their washing in their already overcrowded dwellings. In such dwellings cupboards for food or coal are generally absent, with the result that they smell of stale food day and night, and the coal becomes strewn about the floor and helps to keep the place generally dirty. The dust is received into a common chute, the openings into which are situated generally on the landings and in such an inconvenient position that they are often unused, and the tenant throws the refuse out of the window, or, if it does happen to get into the chute, it leaks out at the various landings through which it passes. The writer happened, unfortunately, to be at the bottom of a six-storey chute when some dust was thrown down at the topmost opening, with the result that he got smothered through a defective flap, and was informed that that had been the normal state of affairs for a long time.

### Sanitary Arrangements.

The common w.c.s are generally situated on the landings, and, unless a very strict watch is kept, they seldom have any locks or fastenings on the doors, the pans are frequently stopped up, and the children often aggravate matters by making no attempt to look for a w.c. in working order on some other landing. These same w.c.s, being open to the public, are not unfrequently used as places of refuge for various purposes. These details are unpleasant and painful, but it is to be feared that many people do not realise what many of our block dwellings are like, and such knowledge is necessary before a right understanding can be arrived at of the essentials of a good block of tenements. Fortunately, what is here described is not the fate of all block dwellings with the defective structural details mentioned, for a great deal depends on the supervision.

### A Superintendent's Duties.

With a superintendent keen on keeping his dwellings clean and respectable he can, by making strict rules and seeing that they are kept, and by a careful selection of his tenants and getting rid of the incorrigible ones, often keep them in such a condition that they do not belie their original designation of "model dwellings," but, once a block of this description begins to go down—*facilis descensus Averni*—the descent is rapid, and nothing short of pulling down or remodelling on a scale tantamount almost to demolition will make them really fit for human habitation.

Many other structural defects of these older dwellings could be mentioned, such as building the individual tenements "back to back" or "side to side" in such a manner as to prevent through ventilation, but enough has been said to prove the need of reform, and it will be our more congenial task to point out in the second article the essentials of a good tenement dwelling.

